# The impact of cosmetic breast implants on breastfeeding: a systematic review and meta-analysis

**DOI:** 10.1186/1746-4358-9-17

**Published:** 2014-10-17

**Authors:** Michal Schiff, Charles S Algert, Amanda Ampt, Mark S Sywak, Christine L Roberts

**Affiliations:** 1 Clinical and Population Perinatal Health Research, Kolling Institute, University of Sydney, Sydney, New South Wales, Australia; 2 University of Sydney Endocrine Surgery Unit, Sydney, New South Wales, Australia; 3 Department of Endocrine and Oncology Surgery, Royal North Shore Hospital, Sydney, New South Wales, Australia

**Keywords:** Breastfeeding, Breast implants, Mammoplasty, Systematic reviews, Meta-analysis

## Abstract

**Background:**

Cosmetic breast augmentation (breast implants) is one of the most common plastic surgery procedures worldwide and uptake in high income countries has increased in the last two decades. Women need information about all associated outcomes in order to make an informed decision regarding whether to undergo cosmetic breast surgery. We conducted a systematic review to assess breastfeeding outcomes among women with breast implants compared to women without.

**Methods:**

A systematic literature search of Medline, Pubmed, CINAHL and Embase databases was conducted using the earliest inclusive dates through December 2013. Eligible studies included comparative studies that reported breastfeeding outcomes (any breastfeeding, and among women who breastfed, exclusive breastfeeding) for women with and without breast implants. Pairs of reviewers extracted descriptive data, study quality, and outcomes. Rate ratios (RR) and 95% confidence intervals (CI) were pooled across studies using the random-effects model. The Newcastle-Ottawa scale (NOS) was used to critically appraise study quality, and the National Health and Medical Research Council Level of Evidence Scale to rank the level of the evidence. This systematic review has been registered with the international prospective register of systematic reviews (PROSPERO): CRD42014009074.

**Results:**

Three small, observational studies met the inclusion criteria. The quality of the studies was fair (NOS 4-6) and the level of evidence was low (III-2 - III-3). There was no significant difference in attempted breastfeeding (one study, RR 0.94, 95% CI 0.76, 1.17). However, among women who breastfed, all three studies reported a reduced likelihood of exclusive breastfeeding amongst women with breast implants with a pooled rate ratio of 0.60 (95% CI 0.40, 0.90).

**Conclusions:**

This systematic review and meta-analysis suggests that women with breast implants who breastfeed were less likely to exclusively feed their infants with breast milk compared to women without breast implants.

## Background

Since the introduction of silicone gel and saline breast implants for cosmetic enhancement of breast size in the early 1960’s, breast augmentation has become one of the most common plastic surgery procedures worldwide [1]. In 2012, 286,000 women in the U.S. had breast augmentation surgery – an increase of 877% from 1992, when the American Society of Plastic Surgeons began formulating yearly national cosmetic surgical statistics [2]. The majority of women who undergo such surgery do so during their reproductive years [3], despite ambiguity regarding the risks to breastfeeding success associated with breast implants.

Breastfeeding has immediate and longer term nutritional, gastrointestinal, immunological, and neurodevelopmental benefits to the baby, and psychosocial benefits for the mother [4]. World Health Organization recognises that while providing some breast milk to the infant is better than none, exclusive breastfeeding is needed to achieve optimal growth, development, and health for infants [5]. If supplementary formula feeding is initiated, the infant does not receive the full advantages of exclusive breastfeeding and the breastfeeding mother must also engage in a complicated balancing act between maintaining or increasing the existing supply while ensuring the infant receives adequate nourishment. The potential to compromise lactation as a result of breast augmentation is particularly relevant with regards to cosmetic breast surgery, which is an elective procedure motivated by aesthetic appeal, rather than in reconstructive surgery (such as following mastectomy). Since there is an element of choice, women need information about all associated risks, both short and long term, in order to make an informed decision regarding whether to undergo cosmetic breast surgery.

The internet currently serves as a prominent source of medical information for people considering plastic surgery [6,7]. However, a considerable amount of the information accessed through search engines regarding breast augmentation in general and its effects on lactation in particular is either misleading or inaccurate [8,9]. Other media have also been shown to be unbalanced, with two thirds of the feature articles on cosmetic surgery in the UK portraying it as risk-free with no mention of potential problems or complications [10]. With the abundance of very accessible, unfiltered sources of information, there is a need for evidence based evaluation of the risk to future breastfeeding ability that can be offered to women considering breast augmentation. The aim of this systematic review is to assess breastfeeding outcomes among women with bilateral cosmetic breast augmentation (also referred to as breast implants, mammoplasty and mammaplasty) compared to women without breast surgery [11]. Specifically to assess 1) the rate of any breastfeeding and 2) among women who breastfeed, the rate of exclusive breastfeeding.

## Methods

### Search methods

A systematic search of published studies in Medline, PubMed, CINAHL and Embase databases using earliest inclusive dates through December 2013 was employed. The search strategy combined terms related to breast surgery along with terms related to breastfeeding, using both subject headings and key words when applicable. There were no language or any other restrictions. The specific search strings used for each of the databases is given in Table 1. The database search was supplemented by hand-searching reference lists of relevant publications.

**Table 1:** Specific search strings used for each of the databases

**String number**	**Medline**	**Embase**	**PubMed**	**CINAHL**
1	exp breast implant/	Breast Implants/	Breast-surgery	Breast implants
2	breast augmentation/	Breast Implantation/	Breast-implants	Breast augmentation
3	exp breast reconstruction/	exp Mammaplasty/	Breast-implantation	Augmentation mammaplasty
4	exp breast prosthesis/	exp "Prostheses and Implants"/	Breast-prosthesis	Augmentation mammoplasty
5	exp breast surgery/	Breast/su [Surgery]	Mammaplasty	Breast enlargement
6	exp plastic surgery/	Surgery, Plastic/	Mammoplasty	Silicones
7	mammaplasty.mp.	mammaplasty.mp.	Breast-augmentation	Breast reconstruction
8	mammoplasty.mp.	mammoplasty.mp.	Breast-enlargement	Breast surgery
9	breast augmentation.mp.	breast augmentation.mp.	Breast and plastic-surgery	Plastic surgery
10	breast enlargement.mp.	breast enlargement.mp.	1 or 2 or 3 or 4 or 5 or 6 or 7 or 8 or 9	1 or 2 or 3 or 4 or 5 or 6 or 7 or 8 or 9
11	breast surgery.mp.	breast surgery.mp.	Breastfeeding	Breastfeeding
12	1 or 2 or 3 or 4 or 5 or 6 or 7 or 8 or 9 or 10 or 11	1 or 2 or 3 or 4 or 5 or 6 or 7 or 8 or 9 or 10 or 11	Breast feeding	Breast feeding
13	exp breast feeding/	exp Breast Feeding/	Lactation	Lactation
14	exp lactation/	exp Lactation/		11 or 12 or 13
15	breast milk/	breastfeeding.mp.	11 or 12 or 13	10 and 14
16	breastfeeding.mp.	breast feeding.mp.	10 and 15	
17	breast feeding.mp.	lactation.mp.		
18	lactation.mp.	13 or 14 or 15 or 16 or 17		
19	13 or 14 or 15 or 16 or 17 or 18	12 and 18		
20	12 and 19			

### Eligibility criteria and outcomes

Studies comparing women who have undergone breast augmentation to women without prior breast augmentation were eligible for inclusion [11]. The outcomes of interest were 1) breastfeeding rates and, 2) among the women who breastfeed, exclusive breastfeeding at the time of assessment. Exclusive breastfeeding was defined as providing only breast milk (directly from the breast or as expressed breast milk) or as defined by the study. Non-exclusive breast milk feeding included any use of breast milk substitute/formula feeding or insufficient lactation as defined by the study.

### Study selection

The review allowed the inclusion of clinical trials and observational studies (cohort, case-control, or cross-sectional studies), but excluded case series or reports, guidelines, comments or reviews without original data [11]. We also excluded studies of women with breast augmentation subsequent to treatment for breast cancer, studies with a comparison group that comprised women with other types of breast surgery, and those lacking a control group altogether.

### Data extraction

The titles and abstracts of all articles identified from the systematic search were screened. The full-text of potentially eligible articles was reviewed for inclusion by at least two independent assessors. Any disagreements regarding inclusion of particular studies were resolved through discussion. After the final list of studies to be included was established, data on the primary and secondary outcomes were extracted independently by two reviewers using a standard form. Results were compared and any discrepancies were resolved through discussion and/or following consultation with a third reviewer.

### Quality assessment

To assess the risk of bias within the included studies, the Newcastle-Ottawa Scale (NOS) for assessing the quality of non-randomized studies in meta-analyses was utilised [12]. Using this scale, a non-randomized study can be awarded a maximum of nine stars on items related to the selection of the study groups (four stars), the comparability of the exposed and unexposed groups (two stars), and the ascertainment of outcomes of interest (three stars). Prior to the rating process, we tailored the scale to capture potential sources of bias relevant to the included studies by pre-specifying the desired minimum duration of follow up to one month postpartum, as well as identifying the main confounding factors (maternal age, parity, intention to breastfeed, gestation at birth and mode of delivery). As the NOS compares non-randomized studies within study design groups, the strength of the evidence was also ranked on the National Health and Medical Research Council Level of Evidence Scale [13]. Using this scale studies are ranked as Level I Evidence for systematic reviews of randomized controlled trials, II for randomized controlled trials, III-1 for pseudorandomized trials, III-2 for comparative studies with concurrent controls, III-3 for comparative studies without concurrent controls and IV for case series. The included studies were rated independently by three reviewers, the scores and ranks were compared, and any differences in scoring were resolved through discussion.

### Statistical analysis

The rate of any breastfeeding following a birth subsequent to breast augmentation, and the rate of exclusive breastfeeding was calculated from the raw data presented in the included papers. The outcomes were assessed for all women in the studies and in a post-hoc subgroup analysis by incision type. For outcomes from two or more contributing studies, rate ratios (RR) from each study were pooled using a random effects meta-analysis, with trials weighted by their inverse variance [14]. Stata’s “metan” command was used to perform the meta-analyses. The degree of variability across studies was summarized using the I^2^ statistic that estimates the percentage of total variation across the studies that is due to heterogeneity rather than chance [15].

## Results

**Figure 1:**
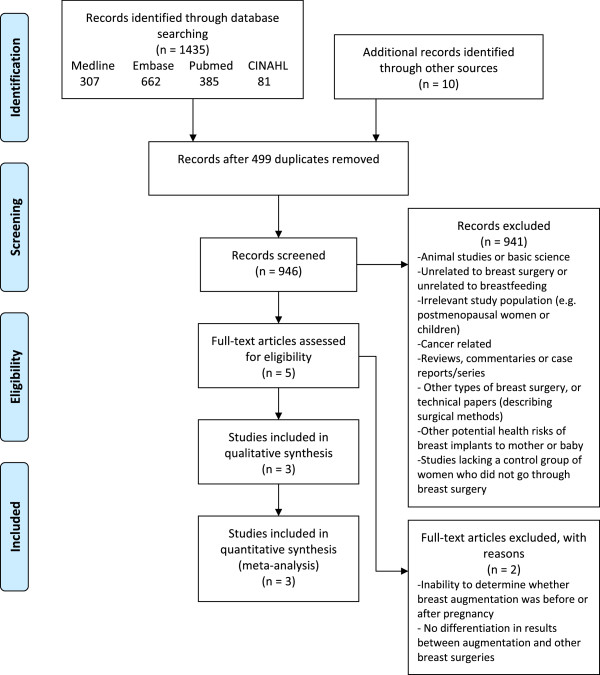
Systematic review flow chart.

Systematic database searches yielded 1435 records, of which 936 were unique citations. A further 10 papers were identified through hand searching. Of 946 unique records, 941 were excluded based on the title and/or abstract as they were irrelevant to the review, did not include the exposure or outcomes of interest, or failed to meet the other stated criteria (Figure 1). Only five full-text articles were reviewed, of which two were excluded due to inability to distinguish pregnancies before and after breast augmentation [16], or between breast augmentation and other breast surgeries [17].

The characteristics of the three included studies are summarised in Table 2. All included studies were hospital-based cohort studies (Evidence Levels III-2 – III-3), enrolling women from either a surgery clinic, a maternity ward, or a lactation support service. Andrade et al. [18] excluded women with more than one type of plastic surgery of the breast, thus not including women with augmentation subsequent to mastectomy, whereas Cruz and Korchin [19] and Hurst [20]’s studies lack any reference to whether women with breast implants for reconstructive purposes were included. While Cruz and Korchin [19] included only women with saline implants in their study cohort, information on implant type is not indicated in the two other studies. Both Cruz and Korchin [19] and Hurst [20], report their findings by the type of incision made for the breast implantation (sub/inframammary or periaerolar). Only one study [18] attempted to reduce confounding by restricting the cohort to ‘healthy’ infants, ‘healthy’ breasts, and mothers without a history of low breast milk production. In contrast, Hurst [20] primarily recruited mothers whose infants were both hospitalized in a children’s hospital and referred to the hospital’s lactation support team. Many of these were high risk babies with high rates of preterm birth and low birth weight. Cruz and Korchin [19] recruited women with small breasts who were evaluated for possible breast augmentation. For women who had previously had children, prior breastfeeding experience was obtained, although the number of children, duration since birth and intention to breastfeed were not reported. Breastfeeding outcomes were then compared to those of women who had a birth subsequent to breast augmentation [19].

**Table 2:** Characteristics of the three included studies

**Author, year**	**Location**	**Study period**	**Study design**	**Study population**	**Cases**	**Controls**	**Data source**	**Outcomes, NOS Score and LOE rank**
Hurst [20]	Texas, U.S.A. Lactation support program in a single children’s hospital	1990-1995	Retrospective cohort study	5066 mothers of babies who were admitted or referred (~15% from primary care) to a tertiary children’s hospital lactation program	42 women with implants who attempted breastfeeding	42 women without implants who attempted breastfeeding (matched on year, lactation course, age, parity and breastfeeding experience)	Lactation follow-up records, documenting breastfeeding progress weekly during infant’s hospitalization and every other week after discharge (by phone), until 2-3 months postpartum or until breastfeeding ceased	Exclusive breast milk feeding or insufficient breastfeeding (defined as little or no lactogenesis or low infant growth with exclusive breastfeeding)
								NOS =5
								LOE = III-2
Andrade [18]	Brazil, single maternity hospital	2004-2005	Cohort study	Women giving birth at the hospital and who attempted breastfeeding	24 women with implants	25 women without implants, selected from same floor as cases	Assessment at home	Exclusive and nonexclusive breastfeeding at 1 month
								NOS =6
								LOE = III-2
Cruz and Korchin [19]	Puerto Rico. Presumably a single plastic surgery clinic	12 month period, year not reported	Retrospective cohort study	18-40 year old women with small breasts who were evaluated for possible breast augmentation	105 women with saline implants who subsequently had children	107 women who had children prior to evaluation for implants	Self-administered questionnaire at initial consultation (controls) or at regular follow-up visit (cases)	Attempted breastfeeding; successful breastfeeding for ≥2 weeks, including exclusive and non-exclusive breastfeeding
								NOS =4
								LOE = III-3

NOS Newcastle-Ottawa Scale assessing the quality of nonrandomized studies in meta-analyses [12].

LOE National Health and Medical Research Council Level of Evidence Scale [13].

The quality of the studies was fair (NOS scores 4-6) and the strength of evidence was low (Evidence Levels III-2 – III-3) (Table 2). NOS scores were reduced for deriving the study population from a single hospital or clinic [18-20], incomplete description of how the exposed cohort was identified [18], selection of cases and controls from different time periods that may lead to biases [19], limited attempt to control for potential confounders [19], using a matched design but an unmatched analysis [20], relying on self-report rather than observation for the assessment of breastfeeding [18-20], follow-up duration shorter than one month [19], and lacking information on loss to follow-up [20].

Assessed outcomes differed considerably across studies. While Cruz and Korchin [19] and Andrade et al. [18] chose to define a time point at which the success of breastfeeding was assessed (two weeks and one month, respectively), Hurst [20] evaluated the overall success of lactogenesis and breastfeeding up to 2-3 months postpartum or until breastfeeding ceased. Notably, while Hurst [20] and Andrade et al. [18] explicitly defined breastfeeding as infants receiving breast milk, whether directly from the breast or as expressed milk, it is unclear whether Cruz and Korchin [19] included expressed breast milk when referring to “successful breastfeeding”.

**Figure 2:**
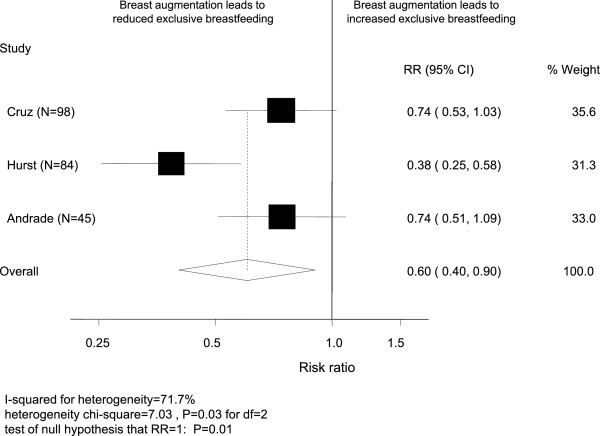
Forest plot of studies that investigated the association between breast augmentation and exclusive breast milk feeding among women who breastfed.

Of the three included studies, only Cruz and Korchin [19] included both women attempting to breastfeed or not, and found similar rates of attempted breastfeeding for women with (59%) and without (63%) breast augmentation (RR 0.94, 95% CI 0.76, 1.17) including 37% and 55%, respectively, reporting any breastfeeding at 2 weeks (RR 0.67, 95% CI 0.50, 0.91). These rates did not differ by incision type. However, among women who breastfed, all three studies [18-20] reported a reduced likelihood of exclusive breastfeeding for women with breast augmentation with a pooled rate ratio of 0.60 (95% CI 0.40, 0.90) (Figure 2). Alternatively, if the outcome is formulated as non-exclusive breastfeeding then the pooled analysis gives a 3-fold increase (RR 3.00, 95% CI 1.16, 7.80) in the use of supplementary formula feeding among women with breast implants who attempt to breastfeed. Of the two studies that examined outcomes by incision type [19,20], sub/inframammary incisions were associated with a reduction in exclusive breastfeeding (pooled RR 0.61, 95% CI 0.46, 0.82) compared to women with breast implants whereas periareolar incisions had a wide confidence interval (pooled RR 0.32, 95% CI 0.04, 2.51) which did not provide evidence of an effect.

## Discussion

Despite the frequency and increasing popularity of breast augmentation [21], this systematic review highlights a lack in the quality and strength of evidence to inform women considering cosmetic breast implants about the potential impact on successful breastfeeding. Although women with breast augmentation were found to be as likely to attempt breastfeeding as women without breast augmentation, women with breast augmentation were less likely to exclusively feed their infants with breast milk. However, the first finding is based on a single study and the second on only three, with none of the included studies having high quality or level of evidence scores [12,13]. Reduced likelihood of exclusive breastfeeding may be attributed directly or indirectly to: the augmentation surgery or the inserted breast implants, an underlying condition (breast hypoplasia), or different attitudes and expectations among women who have breast augmentation surgery.

Breast implantation surgery can cause damage to ducts, glandular tissue, or innervation of the breast [22,23]. Alternatively, breast implants may place pressure on the breast tissue, which can damage the breast tissue or block lactiferous ducts [20]. Reduced capacity to lactate can also result from surgery-related complications [24,25], the most common of which are capsular contracture, hematoma formation, infection, or pain that can turn breastfeeding into a painful experience. The effect of such complications on breastfeeding has been documented in several case studies [26-29]. Risk to lactation capacity increases with time from the initial surgery as some women face the need to undergo reoperation to maintain or improve an initial result, or to treat complications [22]. The studies included in this review did not add to our knowledge of the specific mechanisms by which breast augmentation may disrupt normal breastfeeding function, as there was no detailed information on the surgical history and prevalence of complications was not reported.

Another possible explanation of our findings is the pre-surgical condition of breast hypoplasia, which may be especially prevalent among women choosing breast augmentation. Given current evidence, we are unable to rule out this condition as the cause of reduced milk production and the need to supplement breastfeeding with breast milk substitute. This condition of insufficient glandular tissue - often characterised by small, asymmetrical, or unusually (mostly tubular) shaped breasts, a wide intramammary space and enlarged areolas – can significantly reduce milk production [30]. The incidence of hypoplastic breasts in the general population or its proportion among women choosing to go through breast implantation is unknown. In this regard, Cruz and Korchin’s control cohort of women with previous births who subsequently presented as candidates for breast augmentation may have allowed them to control for pre-surgical conditions [19]. Thus, this study potentially points to the implantation surgery itself, rather than pre-surgical hypoplasia, as the cause of reduced exclusive breastfeeding rates. However, as Cruz and Korchin do not demonstrate the comparability of their cohorts at the time of giving birth (e.g. maternal age, parity, and socio-economic status) [19], differences in the women could also explain the findings.

The observed association of breast augmentation with supplementary feeding could also result from a difference in attitudes and beliefs towards breastfeeding. Women who chose breast augmentation may be more likely to give up breastfeeding once challenged with lactation difficulties, due to prior expectations and lower self-confidence in being able to meet infant’s needs. Alternatively, they may show less perseverance when faced with obstacles due to having a reduced sense of commitment to breastfeed in the first place. Studies of the psychological status of women seeking cosmetic intervention have focused on body image dissatisfaction, low self-esteem and mental health conditions [31-34]. However, attitudes to breastfeeding and their role in preoperative decision making processes and postoperative patient satisfaction, have received little attention. The lack of studies may suggest that maintaining lactation ability is not even part of what most women are concerned with when considering breast augmentation [35]. This may result from the perception of breasts in western culture as sexual, rather than functional organs designed for the feeding of young [36], and is likely exacerbated by advertising that suggests formula and breast milk are equivalent sources for a baby’s nutrition [37-39]. Clarifying the exact reasons for the observed effect requires further research, not only to explore physical causes of reduced breastfeeding capability associated with breast augmentation, but also to elucidate the contribution of psychosocial factors to this intricate picture.

It is problematic to infer no difference in the likelihood of women with breast augmentation attempting to breastfeed based on one small study with a relatively low rate of attempted breastfeeding (59-63%) [19]. Furthermore as this study included only women with saline implants [19], it is possible that the findings do not apply to women with silicone implants. Between 1992 and 2006 the U.S. Food and Drug Administration (FDA) placed silicone gel-filled breast implants in moratorium as a result of serious safety concerns [40,41]. These included concern about the wellbeing of breastfed infants of mothers with silicone gel implants, which was addressed by extensive research aimed at examining the silicone contents of breast milk [42,43] and its implications on infant oesophageal disorders [44-46]. Although no conclusive evidence was found, psychological studies during this period showed that the moratorium and its media coverage had a marked effect on preoperative concerns and postoperative levels of satisfaction of breast augmentation patients [47,48]. It is reasonable to speculate that women with silicone implants who gave birth during the years following the moratorium were less likely to attempt breastfeeding due to hesitance towards the safety of their breast milk [49].

Overall, our systematic search of the literature demonstrated how little has been studied regarding the impact of breast augmentation on breastfeeding outcomes. Surprisingly, although breast implants have a history of more than half a century, and in spite of constant development of new and improved augmentation techniques, only three studies were found to examine this important issue using adequate, no-surgery control groups. These three studies included small cohorts of women, drawn from only a single source, and were based on heterogeneous study populations (Level III evidence) [13]. Based on two studies, we found a reduction in exclusive breastfeeding in the subgroup of women with submammary incisions at augmentation surgery, but could not make a conclusion about those with periareolar incisions. It should be noted that the subgroup analyses were post-hoc and need to be interpreted with caution. Questions related to the implications of implant type (saline vs. silicone) and volume on maintaining breastfeeding capacity have hardly been explored. Further, the three included studies varied in the selected endpoints for assessment of breastfeeding, possibly influencing their ability to capture the difference in breastfeeding course between women with and without breast implants. The heterogeneity across the included studies, along with their moderate scores on the NOS risk of bias assessment, indicates that the effect of breast augmentation may vary depending on maternal characteristics and the need to interpret the pooled estimates with care.

## Conclusions

Our systematic review suggests that breast augmentation is associated with 40% decrease in the likelihood of exclusive breastfeeding among women who breastfeed. However, our finding is based on only three relatively small and heterogeneous studies, and therefore is limited in its external validity. To explore the uncertainty about the observed association and clarify the many unknowns surrounding this issue, more research is required, using larger cohorts and more representative study populations. This information is vital to enable informed decision-making for more than an estimated million women worldwide going through breast implantation surgery each year.

## Competing interests

The authors declare that they have no competing interests.

## Authors’ contributions

CLR and MSS conceived the study and CLR coordinated the project. All authors participated in the study design, planning of analysis and interpretation of the results. CSA undertook the statistical analyses and provided statistical expertise. MS and CLR drafted the manuscript, AA and MSS provided clinical expertise. All authors critically reviewed drafts of the manuscript, and read and approved the final manuscript.
